# Integrative cancer informatics for the identification of prognostic and predictive biomarkers

**DOI:** 10.1186/1753-6561-7-S2-K7

**Published:** 2013-04-04

**Authors:** Igor Jurisica

**Affiliations:** 1 Ontario Cancer Institute and University Health Network, University of Toronto, Canada

## 

Cancer development is a multi-step process that leads to uncontrolled tumor cell growth. Multiple pathways are involved; typically some signalling and regulatory pathways are activated, while others are suppressed. Systematically exploring these networks of proteins will lead to better understanding of disease initiation and progression. Integrating these data with microRNA regulatory networks may identify control mechanism that these master regulators use to affect oncogenesis. Including data on drug targets, modes of actions predicted from drug profiles and compound similarity will in turn lead to more effective patient treatment. To address these challenges, we developed a system for an integrative analysis, prediction and characterization of molecular signatures and relevant protein-protein interactions, microRNA: gene interactions, and resources for rationally identifying drug combinations for cancer treatment.

## Competing interests

There are no competing interests in this presentation.

